# *CTNNA1* hypermethylation, a frequent event in acute myeloid leukemia, is independently associated with an adverse outcome

**DOI:** 10.18632/oncotarget.8962

**Published:** 2016-04-25

**Authors:** Mianyang Li, Li Gao, Zhenling Li, Junzhong Sun, Hui Zhang, Haoqing Duan, Yigai Ma, Chengbin Wang

**Affiliations:** ^1^ Department of Clinical Laboratory, Chinese PLA General Hospital, Beijing, China; ^2^ Department of Hematology, China-Japan Friendship Hospital, Beijing, China; ^3^ Department of Hematology and Oncology, The First Affiliated Hospital of Chinese PLA General Hospital, Beijing, China

**Keywords:** CTNNA1, hypermethylation, acute myeloid leukemia, clinical impact, survival

## Abstract

The aim of this study is to evaluate the frequency of *CTNNA1* hypermethylation in acute myeloid leukemia (AML) patients in an attempt to improve molecular prognostic model. *CTNNA1* promoter methylation levels in 319 newly diagnosed AML patients were detected using quantitative methylation-specific polymerase chain reaction (qMS-PCR). Furthermore, hematological characteristics, cytogenetic abnormalities, and genetic mutation status were analyzed, followed by assessment of clinical impact. Our findings demonstrated that *CTNNA1* hypermethylation was observed in 25% AML patients. Hypermethylation of the *CTNNA1* promoter was associated with unfavorable karyotype, and also possessed the higher frequency of coexisting with *ASXL1* and *RUNX1* mutations. Patients with *CTNNA1* hypermethylation exhibited the shorter relapse-free survival (RFS) and overall survival (OS) in the whole AML and non-M3 AML patients. Moreover, patients with the higher methylation levels had more aggressive course than those with relative lower levels. In multivariate analyses, *CTNNA1* hypermethylation was an independent factor predicting for poor RFS, but not for OS. In conclusion, *CTNNA1* hypermethylation may be a reliable factor for improving prognostic molecular model for AML.

## INTRODUCTION

Recent advances in genetics and epigenetics have improved our understanding of molecular mechanisms of hematological cell transformation and progression to a great extent [1–4]. Numbers of previous studies elaborated leukemic cells exhibited various genetic and epigenetic abnormalities contributing to leukemogenesis, that not only provide the clue for diagnostic stratification and prognostic evaluation, but play a key role for selection of appropriate individuals with suitable targeted therapy [5–8]. Our growing knowledge of the role of aberrant DNA methylation levels silencing leukemia-related anti-oncogenes furnishes a theoretical basis for improving molecular prognostic model [9, 10]. Hypermethylation within the promoters of genes appears to be especially common in some or all types of human hematopoietic neoplasms [11]. Patterns of DNA methylation are nonrandom and tumor-type specific, and this trend has also been shown in acute myeloid leukemia (AML). To date, many genes have been shown to contribute to leukemogenesis through epigenetic silencing. Our previous reports elaborated that aberrant methylation levels of the *CHFR* gene has been detected in acute myeloid leukemia (AML) [12], and *miR-193a* hypermethylation has been discovered for participating in the occurrence of t(8;21) AML [13].

In recent years, a few studies have shown that *CTNNA1* is expressed in normal hematopoietic stem cells (HSCs); however, its expression is significantly lower in human leukemia initiating cells (LICs) in AML [14–16]. Loss-of-function mutations or decreased *CTNNA1* levels have been reported in human cancer cell lines derived from various solid tumors to myeloid malignancies and have been shown to closely be involved in disease progression [17]. This suggests that *CTNNA1* is a promising and essential tumor-suppressor gene involved in leukemic cell transformation, and then give a reasonable presumption that any biomedical procedure that could restore *CTNNA1* expression represents a potential targeted therapeutic strategy to bring benefit to patients with myeloid malignancies.

In the present work, we examined methylation levels of the *CTNNA1* promoter using quantitative methylation-specific PCR method in bone marrow samples from 319 AML patients, with the aim of identifying a subset of patients who harbored aberrant methylation levels and comparing the clinical characteristics of these patients. We also sought to examine chromosome abnormalities and gene mutations associated with AML, for finding significant associations with *CTNNA1* hypermethylation. Furthermore, with the purpose of predicting clinical impact, we would analyzed the relapse-free survival (RFS) and overall survival (OS) according to *CTNNA1* methylation levels.

## RESULTS

### Analysis of DNA methylation levels and gene expression of the *CTNNA1* gene in patients with AML

**Figure 1:**
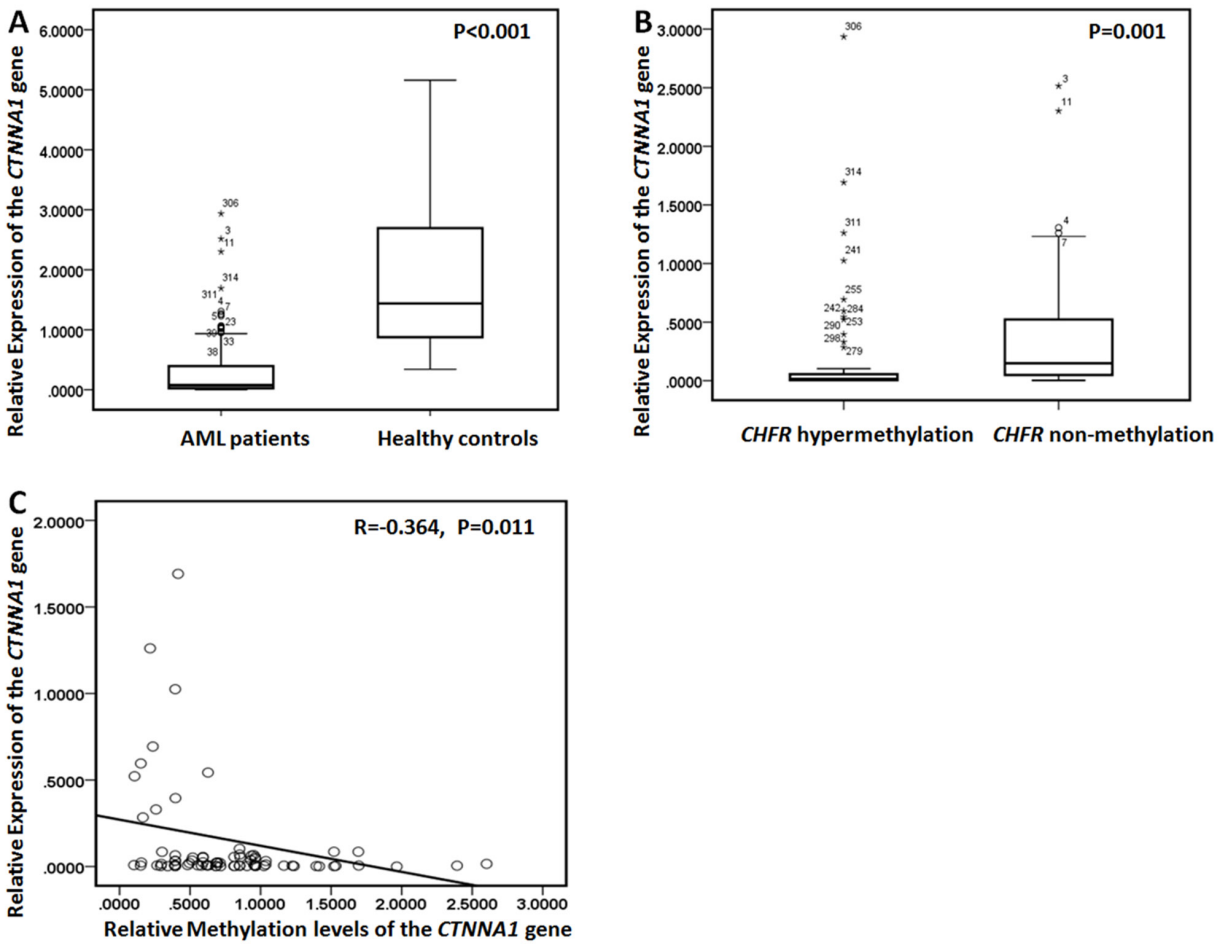
*CTNNA1* mRNA levels and methylation levels in AML patients **A.** Relative expression of the *CTNNA1* gene in 319 AML patients and 30 healthy controls. AML patients exhibited the lower *CTNNA1* mRNA levels than healthy controls. **B.** Relative expression of the *CTNNA*1 gene was detected in the patients with *CTNNA1* hypermethylation and the cases with non-methylation. Patients with *CTNNA1* hypermethylation exhibited lower mRNA transcript levels than those with non-methylation **C.** There was a negative correlation between *CTNNA1* methylation levels and *CTNNA1* transcripts levels (R=−0.364, *P*=0.011).

Promoter methylation levels of *CTNNA1* were assessed in bone marrow samples from 319 AML patients and 30 healthy donors using quantitative methylation-specific PCR (qMSP). *CTNNA1* was hypermethylated in 25% (79/319) AML, but not in samples from healthy donors. In positive patients, the media level was 0.8051 (range, 0.1026–3.3691). Gene expression analysis showed significantly decreased expression of *CTNNA1* in samples of AML patients compared to control individuals (Figure 1A, *P*<0.001). Moreover, there was a significant difference of *CTNNA1* mRNA levels between the patients with hypermethylation and that with non-methylation (Figure 1B, *P*=0.001). Seventy nine patients with *CTNNA1* hypermethylation exhibited lower mRNA transcripts than those with non-methylation. Furthermore, as shown in Figure 1C, among those patients with hypermethylation, *CTNNA1* methylation levels were negatively correlated with mRNA levels (R=−0.364, *P*=0.011).

### Aberrant DNA methylation of the *CTNNA1* promoter was confirmed by bisulfite sequencing

**Figure 2:**
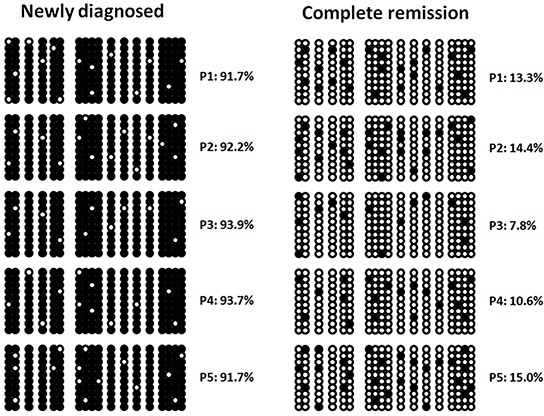
Hypermethylation of the *CTNNA1* promoter in five AML patients by bisulfate sequencing at different clinical stages Methylation rates decreased during hematological complete remission.

In five newly diagnosed AML patients with *CTNNA1* hypermethylation, the promoter of the *CTNNA1* gene was confirmed by bisulfite sequencing with methylation rates of 91.7%, 92.2%, 93.9%, 93.7% and 91.7%, respectively (Figure 2). After traditional chemotherapy employed at our institutions, the methylation rate decreased to different degree in all five patients achieving complete hematological remission with methylation rates of 13.3%, 14.4%, 7.8%, 10.6%, 15.0%, respectively.

### The patients with *CTNNA1* hypermethylation had lower complete remission rate and inferior survival rate

To assess clinical impact of *CTNNA1* hypermethylation, we analyzed clinical characteristics between the patients with DNA hypermethylation and that of without. As Table 1 shown, there were not significant differences in age, sex, white blood cell (WBC), hemoglobin, platelet and marrow blast. The patients with *CTNNA1* hypermethylation had the lower complete remission rate after 1-round chemotherapy. However, 1-year relapse rate and 1-year OS exhibited no differences between the two group. Notably, the cases with *CTNNA1* hypermethylation had the lower 5-year OS rate (*P*=0.001).

**Table 1:** The analysis of clinical characteristics and outcome in two groups

	Total	Hypermethylation (n)	Non-methylation (n)	*P*-value
Patients(N)	319	79	240	NS
Age	40.05 (12-91)	37.27(14-69)	40.97(12-91)	0.085
Sex	186/133	45/34	141/99	0.780
M0	10	4	6	0.216
M1	7	2	5	0.551
M2	84	20	64	0.813
M3	30	4	26	0.128
M4	77	19	58	0.980
M5	90	24	66	0.666
M6	12	3	9	0.606
M7	9	3	6	0.393
WBC(*109/L)	21.91(1.9-83.1)	22.36(2.8-83.1)	21.76(1.9-76.0)	0.740
Hemoglobin(g/L)	76.37(31-131)	76.14(34-131)	76.45(31-131)	0.914
Platelet(*109/L)	40.07(5-146)	39.34(6-141)	40.30(5-146)	0.802
Marrow blast(%)	69.07(50-98)	67.73(50-98)	69.51(50-98)	0.298
Induction therapy				
Decitabine	49	11	38	0.703
DA	134	33	101	0.961
MA	136	35	101	0.729
Allo-HSCT	56	12	44	0.524
Auto-HSCT	31	6	25	0.463
CR rate (1-round therapy)	266/319	59/79	207/240	0.017
1-year survival	302	76	226	0.682
5-year survival	96	12	84	0.001

FAB, French-American-British Cooperative Group; WBC, white blood cell; DA, daunorubicin and cytarabine; MA, mitoxantrone and cytarabine; allo-HSCT, allogeneic hematopoietic stem cell transplantation; auto-HSCT, autologous hematopoietic stem cell transplantation; CR, complete remission.

### DNA hypermethylation of the *CTNNA1* gene was significantly associated with unfavorable karyotype

To further investigate cytogenetic abnormalities in AML patients with and without aberrant methylation levels of the *CTNNA1* gene, we analyzed the differences of various karyotype abnormalities between the two groups. As Table 2 shown, patients harboring *CTNNA1* hypermethylation demonstrated the higher frequency of unfavorable karyotype (*P*=0.010). Furthermore, *CTNNA1* hypermethylation was found to coexist more frequently with +8, −5/5q- compared to non-methylation (*P*=0.006, *P*=0.014, respectively). Among 79 individuals with *CTNNA1* hypermethylation, eight patients harbored −5/5q-, while only 7 patients were detected with chromosome 5 abnormalities in the lower methylation group. Additionally, among 31 patients carrying +8 abnormalites, Fourteen patients had *CTNNA1* hypermethylation, however, 17 cases with non-methylation of the *CTNNA1* gene.

**Table 2:** Comparison of genetic alterations between patients with acute myeloid leukemia with or without hypermethylation of the *CTNNA1* Promoter

Variant	Total (n)	Hypermethylation (n)	Non-methylation (n)	*P*-value
Cytogenetic risk^a^				
Favorable	56	13	43	0.767
Intermediate	172	39	133	0.349
Unfavorable	75	27	48	0.010
Cytogenetic characteristics^b^				
t(8;21)	26	6	22	0.668
t(15;17)	20	2	18	0.088
inv(16)/t(16;16)	14	4	10	0.736
11q23 abnormalities	20	8	12	0.090
+8	31	14	17	0.006
-5/5q-	15	8	7	0.014
-7/7q-	22	6	16	0.778
-X	34	11	23	0.278
-Y	38	9	29	0.869
Complex karyotype	32	10	22	0.370
Normal karyotype	171	40	131	0.541
Gene mutations^c^				
*IDH*	26	6	20	0.835
*ASXL1*	33	15	16	0.001
*FLT3*	28	9	19	0.344
*KIT*	10	3	7	0.493
*TP53*	16	5	11	0.360
*TET2*	30	7	23	0.849
*UTX*	9	3	6	0.393
*SF3B1*	20	5	15	0.580
*CEBPA*	14	2	12	0.282
*MLL-PTD*	15	6	9	0.138
*EZH2*	11	4	7	0.572
*DNMT3A*	26	10	16	0.091
*NPM1*	46	3	43	0.002
*NRAS*	16	3	13	0.409
*SRSF2*	14	6	8	0.103
*SETBP1*	15	4	11	0.533
*RUNX1*	26	14	12	<0.001

a Cytogenetic abnormalities were grouped according to published criteria adopted by Southwest Oncology Group (SWOG) as favorable, intermediate, and unfavorable. Favorable : inv(16)/t(16;16)/del(16q), t(15;17) with/without secondary aberrations, t(8;21) lacking del(9q) or complex karyotypes; Unfavorable: del(5q)/−5, del(7q)/−7, abnormalities of 3q,9q, 11q, 20q, and 17p, t(6;9), t(9;22) and complex karyotypes; Intermediate: normal karyotype, other abnormalities.

b Patients may be counted more than once because of coexistence of more than one cytogenetic abnormality in the leukemic clone.

c Patients may be counted more than once because of coexistence of more than one mutation in the leukemic clone.

### Patients with *CTNNA1* hypermethylation had the higher frequencies of *ASXL1* and *RUNX1* mutations

**Figure 3:**
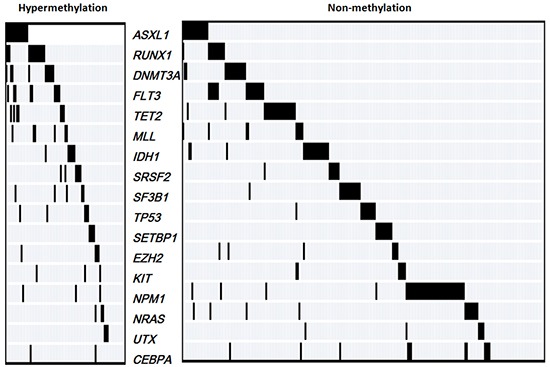
The spectrum of gene mutations in 319 AML patients with hypermethylation and non-methylation of the *CTNNA1* gene

*IDH1*, *ASXL1*, *FLT3-ITD*, *MLL-PTD*, *CEBPA*, *NRAS*, *TET2*, *DNMT3A*, *KIT*, *TP53*, *UTX*, *SF3B1*, *SRSF2, SETBP1, RUNX1* and *EZH2* mutations were detected in 319 individuals with AML using DNA sequencing. The spectrum of mutation status in the patients with *CTNNA1* hypermethylation or non-methylation was shown in Figure 3. The individuals with aberrant *CTNNA1* methylation levels had more probabilities of harboring *ASXL1* and *RUNX1* mutations (*P*=0.001, and *P* <0.001 respectively). In the present work, thirty three patients harbored *ASXL1* mutations, including 15 cases in hypermethylation group and 16 in non-methylation group. Moreover, among 26 patients with *RUNX1* mutations, 14 individual demonstrated aberrant methylation levels of the *CTNNA1* gene.

### Patients harboring aberrant *CTNNA1* methylation levels had poor survival

**Figure 4:**
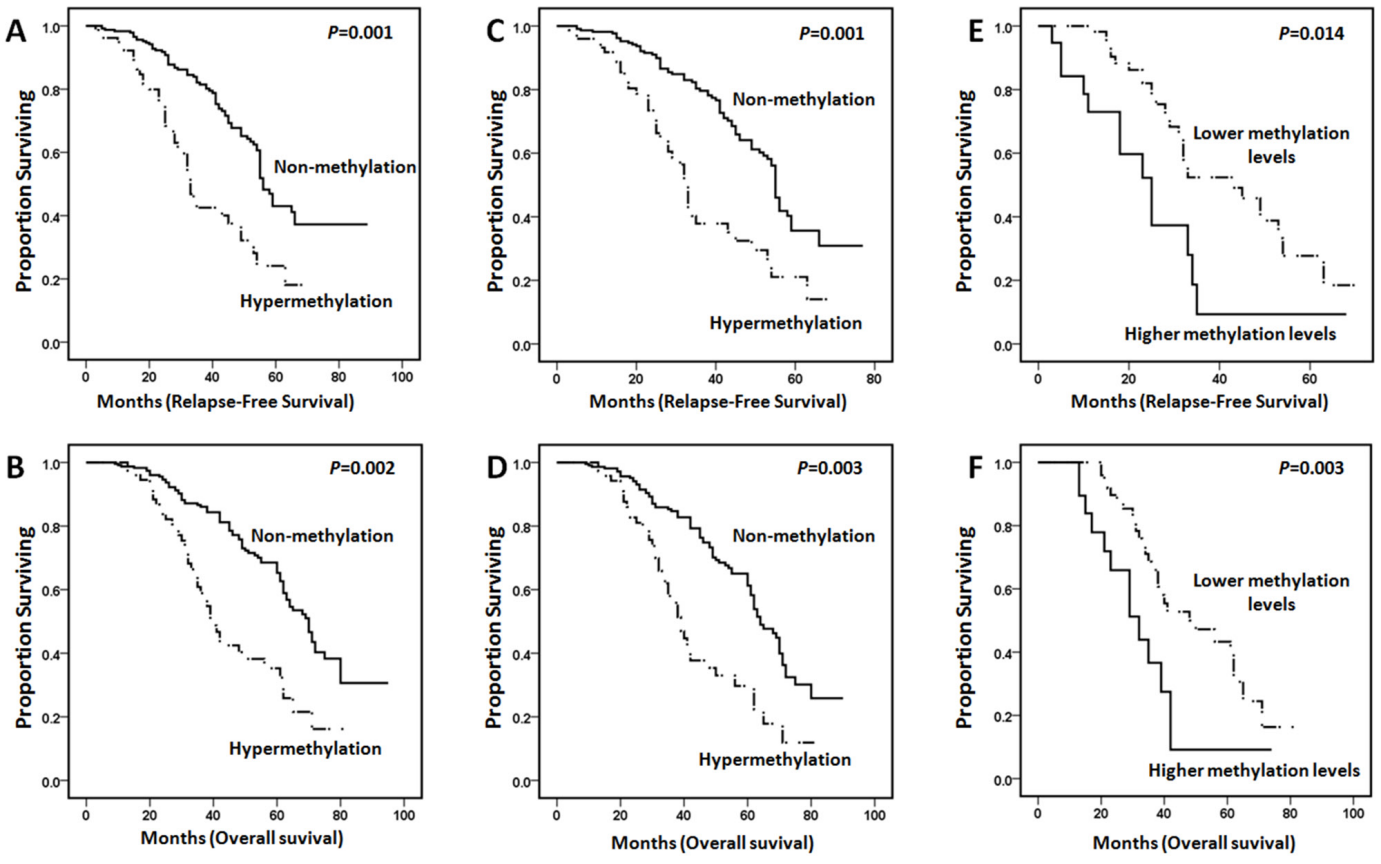
Kaplan-Meier curves for poor relapse-free survival (RFS) and overall survival (OS) in AML patients revealed that *CTNNA1* hypermethylation indicated the shorter survival **A.** and **B.**, In all 319 AML patients, the cases with *CTNNA1* hypermethylation (n=79) had poor RFS and OS compared to those with non-methylation (n=240) (*P*=0.001 and *P*=0.002, respectively). **C.** and **D.**, In non-M3 AML patients, the cases with *CTNNA1* hypermethylation (n=75) had inferior RFS and OS compared to those with non-methylation (n=220) (*P*=0.001 and *P*=0.003, respectively). **E.** and **F.** Patients with higher *CTNNA1* methylation levels (n=19) had adverse RFS and OS compared to individuals with lower methylation levels (n=60) (*P* = 0.014 and *P*=0.003, respectively).

In the present study, RFS and OS were analyzed in two groups with *CTNNA1* hypermethylation and non-methylation. Kaplan-Meier curves indicated that the patients with aberrant *CTNNA1* methylation levels were found to present reduced RFS and OS in all 319 AML patients (Figure 4A and 4B, *P*=0.001, *P*=0.002, respectively). Moreover, the same significant differences were also detected in non-M3 AML about RFS and OS (Figure 4C and 4D, *P* =0.001, *P*=0.003, respectively). To further appraise the prognostic impact of different *CTNNA1* methylation levels, the patients with *CTNNA1* hypermethylation were divided into two groups according to the 75^th^ percentile of the initial methylation levels. Nineteen patients were assigned to the group with the higher methylation levels and the others were assigned to the group with lower methylation levels group. We found that the patients with higher *CTNNA1* methylation levels exhibited inferior RFS (*P*=0.014) and OS (*P*=0.003) (Figure 4E and 4F).

Hypermethylation of the *CTNNA1* gene was entered into a multivariate model in addition to factors significantly associated with prognosis in univariate analysis in our cohort (age>60 years, unfavorable karyotype, *ASXL1* mutation, *SRSF2* mutation, *FLT-ITD*, *RUNX1* mutation, *DNMT3A* mutation, and *MLL-PTD*). In multivariate analysis, *CTNNA1* hypermethylation was an independent factor predicting for poor RFS, but not for OS (Table 3). Furthermore, *DNMT3A* mutation predicted poor RFS independently. The cases with the age of more than 60 years, unfavorable karyotype, *RUNX1* mutation, and *SRSF2* mutation conferred worse RFS and OS in AML.

**Table 3:** Univariate and multivariate analysis of clinical and molecular variables for RFS and OS in AML patients

Variables	Univariate analysis				Multivariate analysis			
	RFS		OS		RFS		OS	
	*P*	OR(95%CI)	*P*	OR(95%CI)	*P*	OR(95%CI)	*P*	OR(95%CI)
age^a^	0.005	0.731 (0.587-0.911)	0.009	0.745 (0.597-0.929)	0.001	0.650 (0.513-0.824)	0.002	0.669 (0.528-0.847)
Unfavorable karyotype^b^	0.014	0.620 (0.423-0.907)	0.018	0.633 (0.433-0.925)	0.016	0.618 (0.418-0.914)	0.022	0.634 (0.429-0.937)
*CTNNA1* hypermethylation	0.001	0.623 (0.515-0.752)	0.002	0.631 (0.522-0.762)	0.034	0.771 (0.606-0.981)	0.060	0.793 (0.623-1.010)
*ASXL1* mutation	0.001	0.649 (0.515-0.818)	0.001	0.636 (0.504-0.803)	0.093	0.790 (0.600-1.040)	0.078	0.780 (0.592-1.028)
*FLT3*-*ITD*	0.004	0.678 (0.521-0.883)	0.004	0.676 (0.520-0.880)	0.051	0.759 (0.577-1.000)	0.055	0.763 (0.578-1.006)
*RUNX1* mutation	<0.001	0.494 (0.376-0.649)	<0.001	0.489 (0.371-0.643)	0.012	0.661 (0.479-0.914)	0.016	0.662 (0.474-0.925)
*MLL-PTD*	0.003	0.628 (0.461-0.857)	0.002	0.617 (0.452-0.841)	0.291	0.820 (0.567-1.185)	0.243	0.799 (0.548-1.165)
*DNMT3A* mutation	0.008	0.670 (0.499-0.899)	0.007	0.664 (0.495-0.892)	0.048	0.725 (0.528-0.997)	0.060	0.734 (0.532-1.013)
*SRSF2* mutation	0.003	0.622 (0.455-0.851)	0.004	0.632 (0.462-0.863)	0.029	0.690 (0.493-0.964)	0.041	0.704 (0.503-0.985)

OR, odds ratio; 95% CI, 95% confidence interval; *FLT3-ITD*, *FLT3* internal tandem duplication; *MLL-PTD*, *MLL* partial tandem duplication; RFS, relapse-free survival; OS, overall survival.

a The patients with the age of more than 60 years vs others.

b Unfavorable cytogenetics versus others.

## DISCUSSION

DNA hypermethylation, which causes transcriptional repression, has recently emerged as one of the most frequent changes occurring in cancers, and has been associated with malignant transformation, making it an intriguing new molecular marker for risk stratification [18]. The use of irreversible DNA methyltransferase inhibitors appears to be a promising option for treatment [19–21]. *CTNNA1* hypermethylation in AML patients has been reported over the years, however the associations with expected gene aberrations that are frequently detected in myeloid malignancies, as well as clinical impact, has not been elaborated [16].

With regard to our findings, we concluded that DNA hypermethylation of the *CTNNA1* promoter was a frequent genetic event in AML that may be a hopeful marker for molecular diagnosis. We confirmed similar results by using bisulfite sequencing at the time of the initial diagnosis and reduced after hematological complete remission in five patients, indicating that *CTNNA1* hypermethylation accounting for functional genetic abnormality may contribute to leukemic transformation. However further studies that aberrant methylation levels of the *CTNNA1* promoter contributing to leukemogenesis need to execute future.

In our series, we adopted qMSP for detecting *CTNNA1* hypermethylation, that being the first report to study methylation levels of the *CTNNA1* gene. The patients with aberrant methylation levels demonstrated the lower 1-year complete remission rate and 5-year overall survival rate, indicating that *CTNNA1* hypermethylation may be an anticipated molecular marker for molecular prognosis. Meanwhile, close associations were found between *CTNNA1* hypermethylation and unfavorable karyotypes, which are recognized as poor markers in AML [22–25]. These findings further suggested that *CTNNA1* hypermethylation could be further considered a novel probable prognostic marker for AML.

In the present study, mutation status were in addition examined in cases with *CTNNA1* hypermethylation and non-methylation. It was worth pointing that *ASXL1* and *RUNX1* mutations were more frequently present in patients with *CTNNA1* hypermethylation. Recent studies demonstrated that *ASXL1* or *RUNX1* mutations were reliable markers indicating for inferior outcome in AML [26–28]. However, the mechanism of conceivable connection of *CTNNA1* hypermethylation with *ASXL1* or *RUNX1* mutations is not clear, that would prompt deep researches. It is to be observed that with the frequent detection of these mutations in our patient groups, a broader view may be further admitted that epigenetic regulation, RNA editing and maturation, as well as transcript factors regulation of hematological development have complex connections that contribute to occurrence and progression of leukemia.

Notably, patients with *CTNNA1* hypermethylation were investigated having poor survival compared with those with non-methylation. In addition, our comprehensive analysis of *CTNNA1* methylation copy numbers provided insights into our understanding of clinical findings in AML patients with different methylation levels. AML patients with the higher methylation levels had inferior RFS and OS, that was the first report that assessment of correlation between methylation copy number and clinical outcome. In multivariate analyses, *CTNNA1* hypermethylation indicated shorter RFS, but not OS, in the entire AML cohort. It is worth mentioning that Chen XX et al demonstrated that hypermethylation of *CTNNA1* promoter was not an adverse prognostic factor for OS in acute myeloid leukemia using methylation-specific PCR [16]. In the present study, qMSP was used for *CTNNA1* methylation levels, that may be one main reason for having a positive survival difference about RFS. Besides, we selected AML patients with more than 50% blasts in bone marrow mainly for reducing experimental error. As for those samples with less than 50% blasts in bone marrow, selection of marrow aspiration site, specimen transporting way, frozen storage time, repeated freezing and thawing cycles may bring immeasurable error for assay data. Thus, that could be another reason for finding decreased RFS in cases with *CTNNA1* hypermethylation.

Recent advances in the field of epigenetic regulations have revealed aberrant methylation levels of gene promoter in addition to abnormal mutation status make leukemogenesis more diversified, thus furnishing more accurate breakthrough for individualized therapy [29, 30]. *CTNNA1*, shown to act as a leukemia-suppressor gene, played a vital role in normal hematological cell development and differentiation [15]. Hence, our analysis of *CTNNA1* methylation levels and associations with clinical, cytogenetic, molecular characteristics revealed important insights into the involvement of prognostic molecular model.

## MATERIALS AND METHODS

### Patient samples

A total of 319 newly diagnosed AML patients and 30 healthy donors attending Chinese PLA General Hospital and China-Japan Friendship Hospital from July 2006 to March 2015 were enrolled in this study. The study was approved by the ethics committees of the participating institutions. Written informed consent was obtained from each subject for sample preservations and genetic analyses. Bone marrow (BM) samples were taken during routine clinical care, and the samples were determined to contain more than 50% blasts by morphologic assessment. Available clinical characteristics were age, sex, French-American-British (FAB) subtype, white blood cell and platelet counts, the amount of BM blasts, and hemoglobin levels. All non-M3 AML patients received intensive induction therapy with DA (daunorubicin and cytarabine) or MA (mitoxantrone and cytarabine) or Decitabine (demethylating treatment) followed by consolidation therapy with cytarabine-based therapy. For M3 patients with t(15;17), all-trans retinoic acid and arsenic trioxide-based treatment was given for induction and consolidation therapy, of which five patients were treated with cytarabine-based therapy as part of consolidation for high-risk diagnosis. Fifty-six patients underwent allogeneic hematopoietic stem cell transplantation (allo-HSCT) and thirty-one cases received autologous hematopoietic stem cell transplantation (auto-HSCT). The clinical characteristics of the patients are described in Table 1.

### Clinical end points

Complete remission (CR) was defined as recovery of morphologically normal BM and blood counts and no circulating leukemic blasts or evidence of extramedullary leukemia. Relapse was defined as 5% or more BM blasts, circulating leukemic blasts, or development of extramedullary leukemia. OS was calculated from date of diagnosis until date of death, censoring patients alive at last follow-up. RFS was calculated from the date of CR until date of relapse or death, regardless of cause, censoring patients alive at last follow-up.

### DNA isolation and bisulfite modification

DNA was isolated from bone marrow using a Genomic DNA Purification Kit (Promega, Madison, WI), and 1 μg of genomic DNA was treated with sodium bisulfite by using a EpiTect Kit (Qiagen, Hilden, Germany) according to the manufacturer's protocols. Modified DNA was resuspended in TE buffer and used immediately or stored at −80°C until use. Bisulfite treatment was used to convert unmethylated cytosines into uracils while leaving the methylated cytosines unaffected.

### Bisulfite sequencing

Bisulfite-treated DNA was amplified with sequencing primers targeting the *CTNNA1* promoter: *CTNNA1*-F, 5′-TTTAGTTTATTTAGAGGAAGTT-3′, and *CTNNA1*-R, 5′-ACTCTCTCAAAACTCCAAAAAAACC-3′. PCR amplified region was shown in Supplementary Figure. Polymerase chain reaction (PCR) products were gel purified and cloned into the pCR2.1-TOPO vector (Life Technologies). Plasmids from single colonies were purified using a QIAprep Spin Miniprep Kit (Qiagen, Germany) and sequenced. Ten cones were selected randomly for sequencing from one patients.

### Quantitative methylation-specific PCR(qMSP)

Bisulfite-treated DNA was amplified using quantitative methylation-specific polymerase chain reaction (qMSP) with *CTNNA1* and *MYOD1* (reference gene) specific primers and probes (Supplementary Table S1). PCR amplified region was shown in Supplementary Figure. Quantitative PCR was carried out in a 40-μL volume with Methylight Master Mix (Qiagen), 0.25 μM appropriate primers and probes, and 20 ng bisulfite-treated DNA. The PCR protocol included 40 cycles of denaturation for 15 second at 95°C and annealing for 60 second at 61°C. A standard curve was produced for *CTNNA1* and *MYOD1* by 10-fold serial dilutions of 5 different plasmid concentrations. The standard curve was saved in a standard curve file. Relative methylation level of *CTNNA1* was calculated by the ratio of copies of *CTNNA1* and *MYOD1.* In all PCR assays, a reference dilution was analyzed, and the standard curve was loaded over this reference sample.

The quantitative range and the sensitivity of the assay were assessed by serially diluting HL60 (fully methylated for *CTNNA1*) into 293 cells (fully unmethylated cell line). Sensitivity was defined as 10^−5^ because it was the lowest dilution with the highest Ct value (Ct 39.2). Furthermore, in the present study, the mean highest Ct value of normal BM was 39.4. In the qMSP assay, *CTNNA1* hypermethylation was defined as a mean Ct value <39.2, and the detectable levels were defined as methylation levels of the *CTNNA1* gene >0.007. The efficiency of our standard curves in all experiments ranged between 95% and 99%. The intra- and interassay coefficients of variation of our qMSP were <0.2.

### Karyotype analysis and fluorescence in situ hybridization (FISH)

Cytogenetic analysis was carried out on BM samples obtained at diagnosis using a direct method or short-term culturing. The cytogenetic reports were reviewed independently by two expert cytogeneticists blinded to patient clinicopathological information. Metaphase chromosomes were banded by G-banding, and chromosomal abnormalities were described according to the International System for Human Cytogenetic Nomenclature [31]. Complex cytogenetic abnormalities were defined as the presence of at least three unrelated cytogenetic abnormalities in one clone. Cytogenetic abnormalities were grouped according to published criteria adopted by Southwest Oncology Group (SWOG) as favorable, intermediate, and unfavorable [32]. Patients with chromosome 5 or 7 abnormalities, inv(16)/t(16;16) and 11q23 abnormalities were confirmed by FISH.

### Real-time quantitative PCR (qPCR)

Bone marrow mononuclear cells were purified by density centrifugation using the standard Ficoll-Hypaque method. Total RNA was isolated from bone marrow mononuclear cells using Qiazol isolation reagent (Qiagen) and was subsequently reverse transcribed to cDNA using a reverse transcription kit (Promega). We performed qPCR to quantify *CTNNA1* transcripts in samples from all patients. The primers and probes specific to *CTNNA1* and *ABL1* are shown in Supplementary Table S2. RT-qPCR was carried out in a 40-μL volume with TaqMan Universal Master Mix (Life Technologies), 0.25 μM appropriate primers and probes, and 20 ng cDNA. The PCR protocol included 40 cycles of denaturation for 15 second at 95°C and annealing for 60 second at 60°C. A standard curve was produced for the *CTNNA1* gene by 10-fold serial dilutions of 5 different plasmid concentrations. The standard curve was saved in a standard curve file. Relative expression of *CTNNA1* was calculated by the ratio of copies of *CTNNA1* and *ABL1*. For all RT-qPCR assays, a reference dilution was analyzed, and the standard curve was loaded over this reference dilution range.

### Detection of gene mutations

*IDH1*, *ASXL1*, *NPM1, FLT3-ITD*, *MLL-ITD, CEBPA*, *NRAS*, *TET2*, *DNMT3A*, *KIT*, *TP53*, *UTX*, *SF3B1*, *SRSF2, SETBP1, RUNX1* and *EZH2* mutations were detected using DNA sequencing for hyper-frequency-mutation sequences as previously reported [33–40], and the primers used for sequencing are shown in Supplementary Table S3.

### Statistical analysis

Statistical analysis was performed using the SPSS 18.0 software (SPSS, Chicago, IL). The chi-square and Fisher's exact (for categorical variables) tests were used to compare patient groups. The correlation between frequency of *CTNNA1* promoter methylation and clinical parameters was analyzed with Pearson's and Spearman's rank correlations. Overall survival curves were plotted using the Kaplan-Meier method and compared using the log-rank test. The median time between visits was 36 months (range: 5 to 100 months). A cox model was used to identify prognostic variables. In addition to *CTNNA1* methylation levels, age, chromosome abnormalities, and mutational status were included as explanatory variables in the regression analyses. For all analyses, *P*-values were two-tailed, and a *P*-value of less than 0.05 was considered statistically significant.

## SUPPLEMENTARY TABLES


