# Supplementary material for: CTNNA1 hypermethylation, a frequent event in acute myeloid leukemia, is independently associated with an adverse outcome
Source: Oncotarget. 2016 Apr 25;7(21):31454–65. doi: 10.18632/oncotarget.8962 (PMC5058770; doi:10.18632/oncotarget.8962)
Supplement: Supplementary file 1 [file oncotarget-07-31454-s001.pdf]

SUPPLEMENTARY TABLES

Supplementary Table S1 : The primers and probes for detection of *MYOD1* and *CTNNA1* methylation levels

Gene	Designation	Sequence(5'-3') and labelling
MYOD1	Forward Primer	GTGATAAAATATTAAATGTGTTTGGTAAGTTTA
MYOD1	Reverse Primer	ATTTTTCTAAAAACTTCCTCAAAACTATCATC
MYOD1	FAM-MGB probe	ATTGTAAAGGTAATTTGATGATAG
CTNNA1	Forward Primer	GAGTTATATGTTTTATTTTCGTATAGTAGGG
CTNNA1	Reverse Primer	AACCAATAAAAACCAAATAACGATC
CTNNA1	FAM-MGB probe	ATTTAGTTTGGTAACGTCG

Supplementary Table S2: The primers and probes used for detecting mRNA levels of the *ABL1* and *CTNNA1* genes

Gene	Designation	Sequence(5'-3') and labelling
ABL1	Forward Primer	AGGCTGCCCAGAGAAGGTCTA
ABL1	Reverse Primer	TGTTTCAAAGGCTTGGTGGAT
ABL1	FAM-MGB probe	TGGAATCCCTCTGACCGG
CTNNA1	Forward Primer	GGGCAATGCTGGACGTAAAG
CTNNA1	Reverse Primer	TCTGAAACGTGGTCCATGACA
CTNNA1	FAM-MGB probe	CACTCAATTCTGCAATAGATA

Supplementary Table S3: The primers used for gene mutation detection

Gene	Forward primer F(5'-3')	Reverse primer R(5'-3')
ASXL1	TGATGCTGCCTCGAGTTGTC<br>AGAAGCTGGGTTTGACCAAAGA<br>CCCCGGCTTGAAGATCGT<br>GGCACCACTTCCTGGGAAA<br>GAGGCCACTAACCCACTTGTG	TTGGTCAAACCCAGCTTCTGT<br>GTGGCTTTTCGGTGTGAACA<br>GTGAGTCCAACTGTAGCCCTCTGT<br>TGCTTCAGAGTCTCCGTTGATTT<br>CCCTTGGCCTGTAACATTGC
DNTM3A	GAAGACCCCTGGAACTGCTACA<br>TCTACCGCCTCCTGCATGAT	GAAGTAGCGGGCCCTGTGT<br>TGGGTGCTGATACTTCTCTCCAT
EZH2	CAGTTCGTGCCCTTGTGTGA	GCCTGGCTGTATCTGTAATCAAAA
FLT3	TCCTGTTTCTCGGATGGATACC	TGGGTCATCATCTTGAGTTCTGA
NRAS	GCCGCATGACTCGTGGTT	GGCGTATTTCTCTTACCAGTGTGTAA
SF3B1	TCAACACTTAGTCCAGAAGAGCAAA<br>GCCATCTTGCCACATCTTAGAAGT<br>TCTTCCTCCCTTTTTTAAACACTTCT	ACTTTCTGCTGCTCATCCACAA<br>CCATCAATCAGTTGTTCTTCAAGTTT<br>GTGGAGTCATCTTATGCATACCTATGA
SRSF2	CGACGCTGAGGACGCTATG	AAGACCTACCCCAAATCCCATT
UTX	CTCTTTGGGTTCGTGAGATTTCA<br>CTTTGTCCAATGCTGAAATTCAAT	AGTGTGATGCATCCAACCTAACTG<br>GGAAGGTTACACAACTGAGCCTGTA
KIT	TACATGGACATGAAACCTGGAGTT	AATGGTCTACCACGGGCTTCT
TP53	CTTGCCGTCCCAAGCAAT<br>CCGAGTGGAAGGAAATTTGC	TGGGCCTCCGGTTCATG<br>GGAGGTAGACTGACCCTTTTTGG
NPM1	AGGAGGAGGATGTGAAACTCTTAAGTAT	AACACGGTAGGGAAAGTTCTCACT
SETBP1	CAGTCACTTGTGGCGTCTTCA	TATGGATTCTCGTGGTAGAAGGTGTA
TET2	AAGCTACTGTGTTTGGTGCG	TCCCAAACTCATCCACGTCA
	CCCACAGAGACTTGCACAAC	TCTGTCTGAGGGTGATGTGG
	CTCAAGCATAACCCACCAATTTT	TGGCCAAAGAATGATCCTTCTC
	GCCCTGCATCTCCAAAACAA	CCCGAGTGAAGGCATATGGA
IDH1	CCAAGTCACCAAGGATGCTG	CCATGTCGTCGATGAGCCTA
CEBPA	AAGAAGTCGGTGGACAAGAACAG	GCAGGCGGTCATTGTCACT
MLL	GTTTAGAGGAGAACGAGCGC	CAAACACCACAGTCCTCAGG
RUNX1	GTCATTTCCTTCGTACCCACAGT	GTGTGGGCTGACCCTCATG
